# Integrative transcriptomic and machine learning analyses identify HDAC9 as a key regulator of mitochondrial dysfunction and senescence-associated inflammation in diabetic nephropathy

**DOI:** 10.3389/fimmu.2025.1627173

**Published:** 2025-08-29

**Authors:** Junming Huang, Dong Pang, Chenglong Fan, Guanglin Yang, Jinji Chen, Shaohua Chen

**Affiliations:** ^1^ Department of Urology, Guangxi Medical University Cancer Hospital, Nanning, Guangxi, China; ^2^ Department of Obstetrics and Gynecology, The People’s Hospital of Guangxi Zhuang Autonomous Region, Guangxi Academy of Medical Sciences, Nanning, China

**Keywords:** diabetic nephropathy, mitochondrial dysfunction, senescence, machine learning, biomarker

## Abstract

**Background:**

Diabetic nephropathy (DN), a major complication of type 2 diabetes mellitus (DM), is driven by complex mechanisms involving mitochondrial dysfunction, senescence, and chronic inflammation. Despite therapeutic advances, interventions specifically targeting mitochondrial dysfunction, senescence, and inflammation remain elusive.

**Methods:**

An integrative analysis was performed on bulk RNA-seq data from DN and normal kidney samples to identify differentially expressed genes (DEGs) associated with the disease. Weighted gene co-expression network analysis (WGCNA) was utilized to reveal gene modules linked to DN, mitochondrial dysfunction, and senescence. The key genes were determined using multiple machine learning approaches, and their diagnostic value was verified using external datasets. At single-cell resolution, the cellular landscape of DN was explored and the distinct expression patterns across different cell types were explored. Key genes and markers associated with mitochondrial dysfunction and senescence were validated through single-cell RNA sequencing (scRNA-seq) data and *in vitro* high-glucose-induced HK-2 cell models. Finally, functional studies were conducted using Small interfering RNA (siRNA)-mediated gene knockdown to predict the biological roles of selected targets.

**Results:**

We identified 2,176 DEGs between DN and normal kidney tissues, among which 259 mitochondrial-related genes (MRGs) and 273 senescence-related genes (SRGs) were significantly enriched in inflammatory and metabolic pathways. WGCNA revealed DN-associated gene modules strongly linked to mitochondrial dysfunction and senescence. Through integrated machine learning, five hub genes—CLDN1, TYROBP, HDAC9, CASP3, and RCN1—were selected, with the support vector machine (SVM) model achieving high diagnostic accuracy. ScRNA-seq revealed 13 distinct kidney cell types, with proximal tubule (PT) cells emerging as key contributors to the signaling pathway associated with mitochondrial dysfunction and senescence. These transcriptomic findings were corroborated by functional assays, in which HDAC9 upregulation in high-glucose-stimulated HK-2 cells was accompanied by mitochondrial impairment and increased levels of p53, p21, p16, and senescence associated secretory phenotype (SASP) factors. Conversely, HDAC9 knockdown mitigated these effects, underscoring its pathogenic role in DN.

**Conclusion:**

Mitochondrial dysfunction and senescence-associated inflammation contribute to DN progression. The five identified hub genes demonstrate strong diagnostic potential, and HDAC9 is likely to be a potential therapeutic target for reducing mitochondrial injury, senescence, and inflammation in DN.

## 1 Introduction

Diabetes mellitus (DM) is a complex metabolic disorder characterized by impaired glucose homeostasis. The global prevalence of DM has been on the rise since 1980, with approximately 10% of adults now affected worldwide (1). Diabetic nephropathy (DN) is a major complication of diabetes with multifaceted pathogenesis, encompassing epithelial dysfunction, glomerular hyperfiltration, hemodynamic perturbations, and inflammatory processes (2–4). Approximately 30-40% of patients are likely to develop DN. However, majority of current therapeutic strategies aim to delay rather than prevent the progression to end-stage renal disease, which underscores the need for novel therapeutic targets to improve patient outcomes (5, 6). Accumulating evidence has demonstrated that abnormalities in key pathways and cellular processes contribute substantially to renal dysfunction in DN (7, 8). Among various pathological changes such as advanced glycation end products (AGEs) accumulation and oxidative stress (9). mitochondrial dysfunction and cellular senescence are pivotal drivers of DN onset and progression.

As a highly metabolic organ with abundant mitochondria, the kidney depends on substantial adenosine triphosphate (ATP) production to maintain its normal physiological functions (10). Mitochondrial energetics are disrupted by hyperglycemia in DN, which leads to increased reactive oxygen species (ROS) generation and reduced ATP synthesis (11). These changes promote mitochondrial fission, alter mitochondrial morphology, and exacerbate disease progression (12). Mitochondrial impairment not only compromises the normal functions of tubular and glomerular cells (13) but also triggers immune activation through the release of damage-associated molecular patterns (DAMPs) (14). These DAMPs may act as endogenous danger signals that activate innate immune cells—including macrophages, dendritic cells, and neutrophils which promotes the secretion of pro-inflammatory cytokines and chemokines. This, in turn, recruits adaptive immune cells such as B and T lymphocytes, thus amplifying renal inflammation (15). The resulting immune cell infiltration drives and sustains inflammatory responses within the kidney, culminating in tubulointerstitial fibrosis and glomerulosclerosis (16, 17). While mitochondrial dysfunction and immune-mediated inflammation play a significant role in DN pathogenesis, the interplay between these mechanisms remains poorly understood and requires further investigation.

Senescence contributes to physiological decline and increased disease susceptibility (18), driven by dysfunction across homeostatic systems (19). Therefore, targeting senescence-associated inflammation is considered a promising strategy for senescence-related therapeutic interventions (20). Senescence plays a critical role in DN (21, 22). A complex interplay of factors, including chronic hyperglycemia, persistent inflammation, and disrupted lipid metabolism, collectively contribute to nephron damage and DN progression (23, 24). This indicates that investigating the biological interplay between mitochondrial dysfunction and senescence within the framework of DN is essential. Bulk transcriptomic analyses have provided valuable insights into both experimental models and human DN (25). However, these approaches are inherently limited by the loss of cell-type-specific signals within the pooled data. Single-cell RNA sequencing (scRNA-seq) can help to address this limitation by generating high-resolution cellular atlases that capture the diversity of cell types and states in physiological and pathological contexts. Given the diverse range of cell types involved in DN, we hypothesized that scRNA-seq may offer a deeper understanding in DN pathogenesis.

The present study explored the molecular mechanisms underlying DN through comprehensive transcriptomic and single-cell analyses. Initially, differentially expressed genes (DEGs) between DN and normal control (NC) samples were identified. Meanwhile, key mitochondrial-related genes (MRGs) and senescence-related genes (SRGs) module were determined via weighted gene co-expression network analysis (WGCNA) and functional clustering. Functional enrichment analysis revealed significant associations among mitochondrial dysfunction, inflammation, and senescence. In addition, diagnostic models were developed using various machine learning algorithms. Moreover, we verified the expression levels of hub genes and investigated the unique communication patterns across different cell types at single-cell resolution using scRNA-seq data. Finally, a high-glucose-induced HK-2 cell model was developed to verify the expression of model genes, as well as marker genes related to mitochondrial dysfunction and cellular senescence. Overall, this study aimed to clarify the role of mitochondrial dysfunction and senescence in the pathogenesis of DN.

## 2 Materials and methods

### 2.1 Data acquisition and sample information

DN datasets were obtained from the Gene Expression Omnibus (GEO) repository (https://www.ncbi.nlm.nih.gov/geo/), encompassing bulk-tissue messenger RNA (mRNA) sequence datasets (GSE30122, GSE30528, GSE30529, GSE96804, and GSE142153). Among these datasets, GSE30529 and GSE142153 served as independent validation sets. This comprehensive compilation included 92 DN samples juxtaposed with 93 NC samples. Furthermore, 2,794 human genes encoding proteins, with robust evidence supporting mitochondrial localization, and 2,001 genes associated with senescence were extracted utilizing GeneCards with a relevance score threshold of 1.0 (https://www.genecards.org/).

### 2.2 Identification of DEGs and DN-related genes (DNRGs)

The merged datasets, namely GSE30122, GSE30528, and GSE96804, comprised the training set. The R package “sva” was used to remove batch effects from a training set. The “limma” R package (*P* < 0.05 and |log2 fold change (FC) | > 0.585) was utilized to identify DEGs in the training set.

WGCNA assumes paramount significance in bioinformatic analysis and is widely adopted for trait and gene association studies. To uncover important DNRGs, we first performed WGCNA based on the expression matrix of the bulk RNA-seq data. Genes located within the module exhibiting the strongest correlation with the DN phenotype were designated as DNRGs, reflecting their close association with DN based on co-expression patterns. These DNRGs were subsequently intersected with DEGs to identify key candidate genes for further analysis. The R package “WGCNA” was employed to construct a co-expression network.

### 2.3 Consensus clustering in the training set

Clustering analysis of the DN sample was conducted by “ConsensusClusterPlus” package, utilizing expression levels of DEGs. The optimal number of clusters was determined by evaluating the consensus matrix, consensus cumulative distribution function (CDF), and trace plots.

### 2.4 Identification of SRGs and MRGs

The MRGs were identified by intersecting genes encoding mitochondrial-related proteins, DNRGs, and DEGs. Similarly, SRGs were identified by intersecting genes encoding senescence related-proteins, DNRGs, and DEGs. To mitigate the high false discovery rates observed in previous studies, the identified genes were subjected to further filtering using the Wilcox test between DN and NC groups (*P* < 0.05). Subsequently, Gene Ontology (GO) and Kyoto Encyclopedia of Genes and Genomes (KEGG) pathway enrichment analyses were performed.

### 2.5 Phenotype scoring of mitochondrial dysfunction and senescence analysis and model establishment

MRGs and SRGs underwent further intersection and screening utilizing four machine learning algorithms to identify key genes. Support Vector Machine (SVM) was implemented using the “kernlab” R package. The random forest (RF) algorithm was implemented using the “randomForest” R package. Additionally, the XGBoost (XGB) algorithm, known for its efficient and scalable implementation of gradient boosting, was implemented using the “xgboost” package.

Mitochondrial dysfunction and senescence phenotype scores were calculated using the single-sample gene set enrichment analysis (ssGSEA) algorithm with the “GSVA” R package. Differences in phenotype scores between groups were assessed, and correlations among phenotype scores were examined. Subsequently, DN and control samples were segregated based on the median phenotype scores for subsequent GSEA involving all genes.

### 2.6 Verification of diagnostic model for DN

The performance of the diagnostic model for DN was assessed using the “pROC” package. Further analyses were conducted to identify the five most important features contributing to the model’s prediction accuracy. Moreover, the model was validated using an independent dataset, GSE142153, to evaluate its robustness and generalizability.

### 2.7 ScRNA−seq analysis

The scRNA-seq dataset GSE183276, comprising 9,235 cells from healthy kidneys and 27,929 cells from the DN cohort, was analyzed using the standard “Seurat” pipeline to assess single-cell characteristics. Cells were filtered based on stringent criteria: those with fewer than 400 genes, more than 5000 total genes, and over 30% mitochondrial genes were excluded. After quality control filtration, the R package “harmony” was employed to mitigate batch effects among samples. Subsequently, cell cluster annotation was performed based on previous research findings. Uniform Manifold Approximation and Projection (UMAP) was employed for visualization. To further clarify the intercellular communication patterns within the kidney microenvironment, we utilized the “CellChat” R package, which infers ligand–receptor interactions and predicts signaling networks across cell types.

### 2.8 Cell culture and treatment

The human renal tubular epithelial cell line (HK-2) (WHELAB C1116) was procured from Shanghai Whelab Bioscience Limited (Shanghai, China). HK-2 cells were cultured in Dulbecco’s Modified Eagle Medium/Nutrient Mixture F-12 (DMEM/F12) (Gibco, China) supplemented with 10% fetal bovine serum (Sigma-Aldrich, China) and 100 U/ml Penicillin-Streptomycin Liquid (Solarbio, Beijing, China) at 37 °C in a humidified incubator with 5% CO2. When the cell density reached 60-70%, glucose solution was added to the complete medium to achieve a final glucose concentration of 40 mmol/L. The cells were then incubated under the same conditions for 72 hours to establish a DN cell model (26). For the control group, an equal volume of phosphate-buffered saline solution was added to the complete medium, and the incubation conditions and duration were similar to those of the DN cell model group. After 72 hours, cells from the DN cell model and the control groups were collected for subsequent experiments.

### 2.9 Small interfering RNA (siRNA) transfection

siRNAs and the transfection reagent RNATransMate (E607402) were obtained from Sangon Biotech (Shanghai, China). HK-2 cells were seeded in six-well plates and cultured for 12 hours to reach a cell density of 60-70%. Next, appropriate amounts of siRNAs and RNATransMate were added to the six-well plates according to the manufacturer’s instructions to achieve a final siRNA concentration of 40 nM. After 4–6 hours of transfection, the medium was replaced with either a complete medium or a medium for constructing the DN cell model, and cells were cultured for an additional 72 hours. Subsequently, these cells were used for reverse transcription-quantitative polymerase chain reaction (RT-qPCR). The primer sequences used are provided in **Supplementary Table 1**.

### 2.10 RT-qPCR

Total RNA was extracted from HK-2 cells using an RNA extraction kit (Magen, Shanghai, China) following the manufacturer’s protocol. The isolated RNA was reverse-transcribed into complementary DNA (cDNA) using the PrimeScript RT Master Mix (Takara, Japan). RT-qPCR was performed using the ABI Applied Biosystems PowerUp SYBR Green Master Mix (Thermo Fisher Scientific, USA) on a LightCycler 96 real-time PCR system (Roche Diagnostics Gmbh, Switzerland). β-actin served as the internal reference gene. Relative gene expression levels were calculated using the comparative cycle threshold (2^-ΔΔCt^) method. The primer sequences used are provided in **Supplementary Table 1**.

### 2.11 Western blot (WB) analysis

HK-2 cells were lysed in radioimmunoprecipitation assay buffer (Beyotime, Shanghai, China) supplemented with a protein phosphatase inhibitor. The lysates were centrifuged at 14,000 × g for 10 minutes to extract total protein. Proteins were separated by sodium dodecyl sulfate-polyacrylamide gel electrophoresis and subsequently transferred onto polyvinylidene fluoride membranes. Membranes were then blocked with 5% Skim milk for the specified time. Subsequently, membranes were incubated at 4°C overnight with primary antibodies, followed by incubation with secondary antibodies at room temperature for 1 hour. The details of the antibodies used in this study are provided in **Supplementary Table 2**. Protein bands were visualized using enhanced chemiluminescent reagents HY CEZMBIO West ECL-A and ECL-B (Hubei, China). The resulting bands were scanned, and their intensities were analyzed and quantified using ImageJ software and normalized to the corresponding internal controls.

### 2.12 Transmission electron microscopy

After reaching a culture density of 70%, HK-2 cells were digested with trypsin and centrifuged at 3,000 × g for 5 minutes. Cells were resuspended in an electron microscopy fixation solution (Servicebio, Wuhan, China) and gently dispersed. Samples were fixed at room temperature for 30 minutes in the dark and then stored at 4 °C. Ultrathin sections were prepared and then stained with 2% uranyl acetate saturated in ethanol in the dark. Finally, the ultrathin sections were visualized under a transmission electron microscope (HITACHI HT7800/HT7700).

### 2.13 Statistical analysis

Differences in continuous variables were assessed using the Wilcoxon test. Pearson correlation analysis is used to assess the correlation between two continuous variables. Data were visualized using the R package “ggplot2”. All statistical tests were two-sided, and *P* < 0.05 was considered statistically significant. Significance levels were denoted as follows: ‘ns’ for not significant, *P < 0.05, **P < 0.01, and ***P < 0.001.

## 3 Results

### 3.1 Identification of DEGs and DNRGs

**Figure 1:**
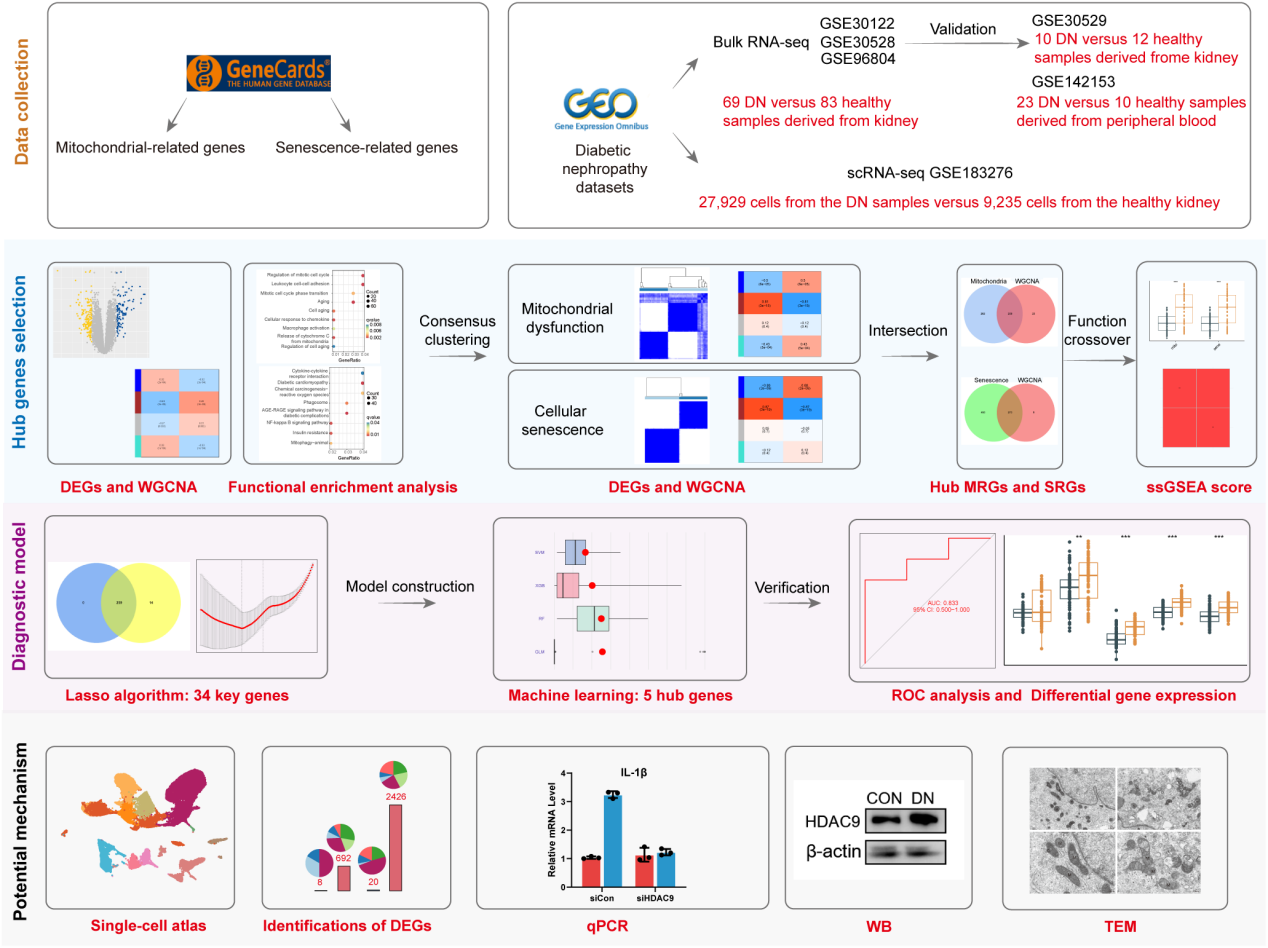
A flow chart of the study.

**Figure 2:**
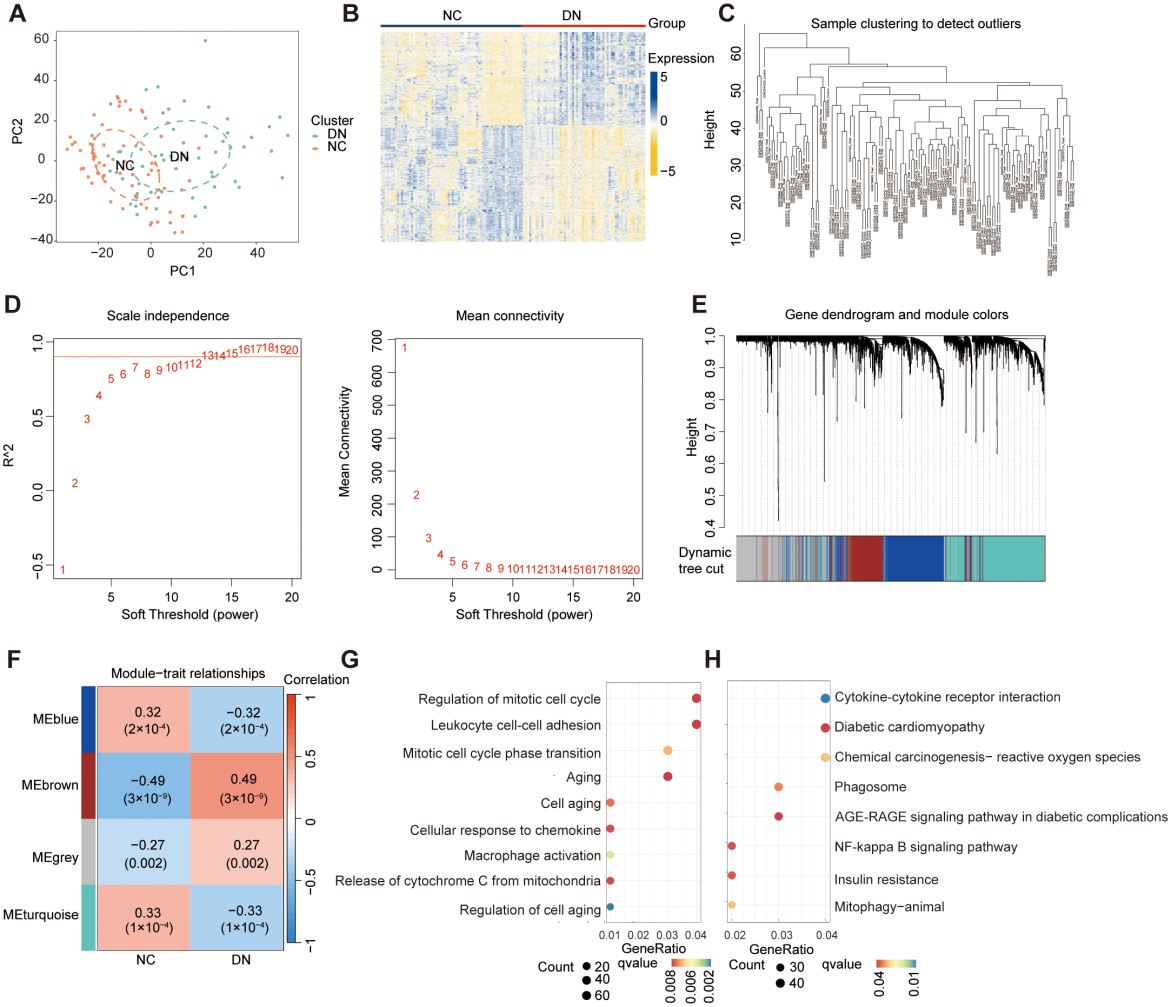
Identification of DEGs and DNRGs. **(A)** The PCA results for the training datasets. **(B)** A heat map of the identified DEGs. **(C)** WGCNA sample clustering. **(D)** Soft-thresholding filtering. **(E)** Clustering dendrogram of genes. **(F)** Correlation heatmap of gene modules and clinical features. **(G)** GO and **(H)** Results of the KEGG enrichment analysis of the DEGs.

The datasets information and workflow of the presented study are shown in **Figure 1**. In this study, we integrated three bulk RNA-seq datasets—GSE30122, GSE30528, and GSE96804—as a combined training cohort for differential gene expression analysis. Principal component analysis (PCA) revealed a clear distinction between DN and NC samples based on the expression profile of the training set (**Figure 2A**). Differential expression analysis revealed 2,176 DEGs between the DN and NC samples (**Figure 2B**). Subsequently, WGCNA analysis was conducted, wherein the sample clustering diagram displayed discernible patterns (**Figure 2C**), and a soft-thresholding power (β) of 14 yielded a scale-free topology with a correlation coefficient exceeding 0.85, supporting robust network construction (**Figure 2D**). Employing the dynamic tree cut algorithm, multiple gene modules were initially identified (**Figure 2E**), subsequently, we determined the correlations between each gene significance (GS) and module membership (MM), and retained four modules with relatively strong associations (GS > 0.2 and MM > 0.6) for further analysis. Among them, the brown module showed a significantly high correlation (R = 0.49) and significant *P* value (*P* = 3 × 10⁻^9^) with DN (**Figure 2F**). Consequently, genes within the brown module were designated as DNRGs. Notably, GO (**Figure 2G**) and KEGG (**Figure 2H**) functional enrichment analyses revealed that the differentially expressed DNRGs were primarily associated with pathways related to mitochondrial dysfunction, cellular aging, insulin resistance, and inflammation. Collectively, these results demonstrate that genes clustered within the brown module were strongly associated with DN and were predominantly involved in mitochondrial dysfunction, senescence, insulin resistance, and inflammatory processes, providing a robust gene set for subsequent biomarker selection and mechanistic investigations.

### 3.2 Unsupervised clustering analysis of DEGs and WGCNA

**Figure 3:**
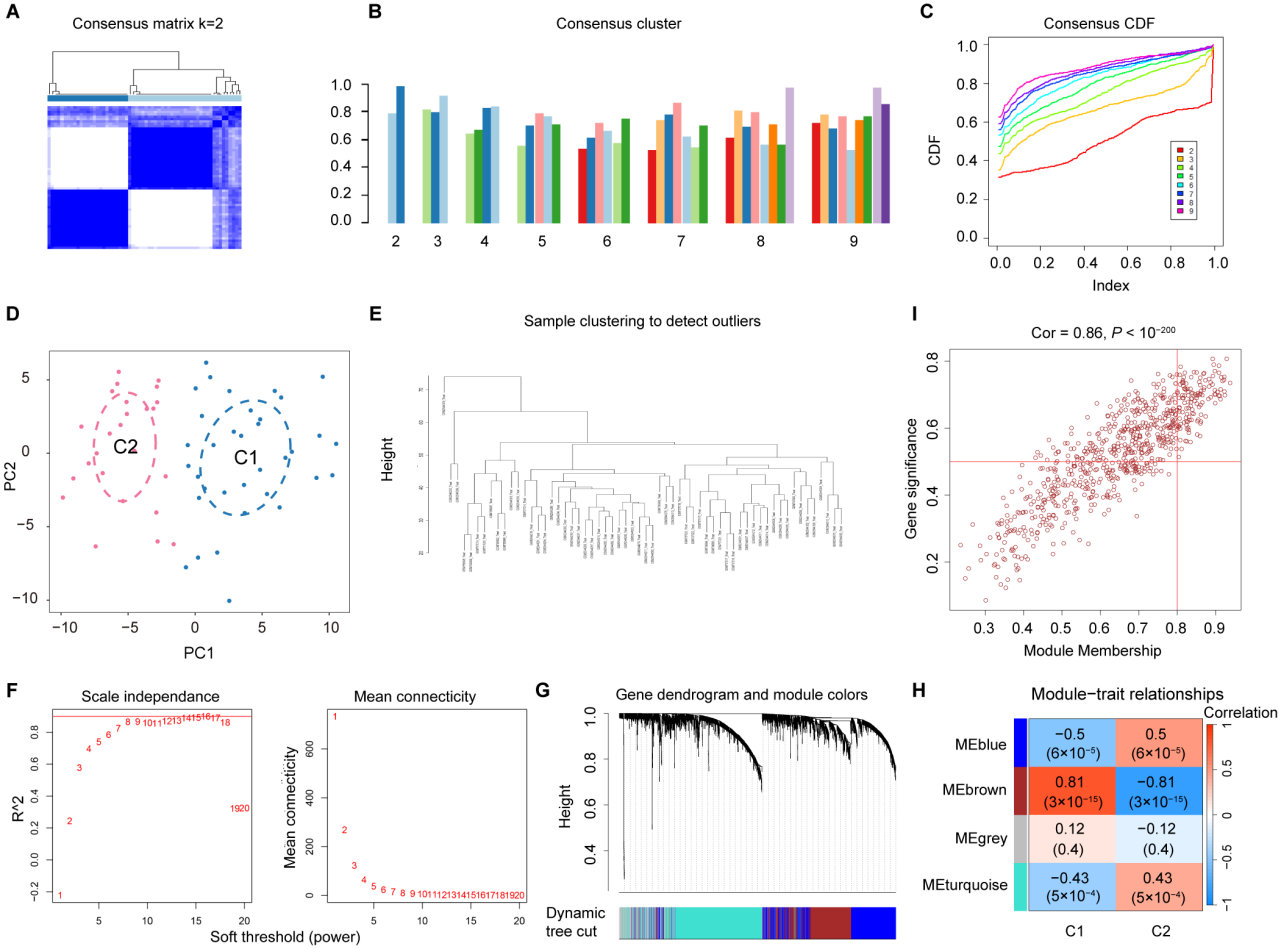
Screening for the MRGs in DN. **(A)** Two clusters identified at k=2 **(B)** Consensus clustering scores for k=2 to 9. **(C)** Consensus clustering CDF for k = 2 to 9. **(D)** PCA showing the distribution of two mitochondrial dysfunction clusters. **(E)** WGCNA sample clustering. **(F)** Soft-thresholding filtering. **(G)** Gene clustering dendrogram. **(H)** Correlation heatmap of the gene modules and clinical features. **(I)** Relevance of the brown module members to DN.

To investigate the diverse expression patterns of DEGs associated with mitochondrial dysfunction in DN, consensus clustering was applied. This analysis identified two distinct clusters, as shown in the consensus matrix (k = 2, **Figure 3A**). Moreover, each cluster showed a score exceeding 0.8 at k = 2 (**Figure 3B**). The robustness of clustering was further confirmed based on minimal fluctuations of the consensus CDF curves across various consensus indexes (**Figure 3C**). Consequently, 60 DN samples were classified into two clusters: Cluster 1 (C1; 29 samples) and Cluster 2 (C2; 31 samples). PCA effectively separated the clusters (**Figure 3D**). WGCNA was performed to identify gene co-expression modules related to DN subtypes. Outlier samples identified through hierarchical clustering were excluded (**Figure 3E**). Following the selection of genes with the top 25% variance and exclusion of abnormal samples, a scale-free network was constructed with a soft threshold of 16, yielding a scale-free R^2^-value of 0.975 (**Figure 3F**). The WGCNA algorithm was employed to explore key genes associated with DN. The sample clustering diagram revealed four distinct co-expression modules (**Figure 3G**).

Among the identified gene modules, the brown module exhibited a significant correlation with mitochondrial dysfunction, with a coefficient of 0.81 and a *P* value of 3 × 10⁻^15^ (**Figure 3H**). In addition, the GS and MM were highly correlated in the brown module (**Figure 3I**). From this module, 461 genes were identified and selected for further analysis. Due to their significant positive association with mitochondrial dysfunction, these genes were designated as MRGs (**Supplementary Table 3**).

**Figure 4:**
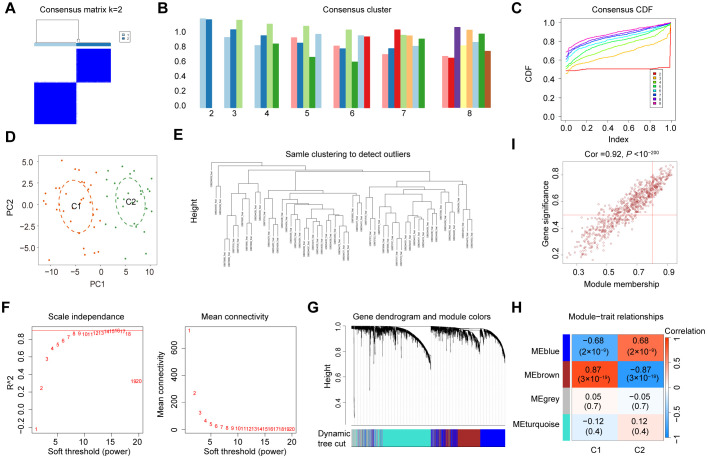
Identification of SRGs in DN. **(A)** Two clusters identified at k=2. **(B)** Consensus clustering scores for k=2 to 9. **(C)** Consensus clustering CDF for k = 2 to 9. **(D)** PCA represents the distribution of two senescence clusters. **(E)** WGCNA sample clustering. **(F)** Soft-thresholding filtering. **(G)** Gene clustering dendrogram. **(H)** Correlation heatmap of the gene modules and clinical features. **(I)** Relevance of brown module members to DN.

To explore the diverse expression patterns of the DEGs associated with senescence in DN, consensus clustering was performed. This analysis identified two optimal clusters (k = 2), as shown in the consensus matrix (**Figure 4A**), with consensus scores exceeding 0.8 (**Figure 4B**) and stable consensus CDF curves (**Figure 4C**). The 60 DN samples were divided into C1 (33 samples) and C2 (27 samples), which were separated by PCA (**Figure 4D**). Outliers were excluded using a sample clustering tree (**Figure 4E**). Genes with the top 25% variance were selected and used to construct a scale-free network through the soft threshold of 16 (**Figure 4F**). The WGCNA test identified four co-expression modules (**Figure 4G**), with the brown module showing the strongest correlation with senescence (coefficient: 0.87, *P* = 3 × 10⁻^19^) (**Figure 4H**). A strong correlation was found between GS and MM in the brown module (**Figure 4I**), which identified 726 SRGs (**Supplementary Table 4**).

### 3.3 Functional overlap between MRGs and SRGs

**Figure 5:**
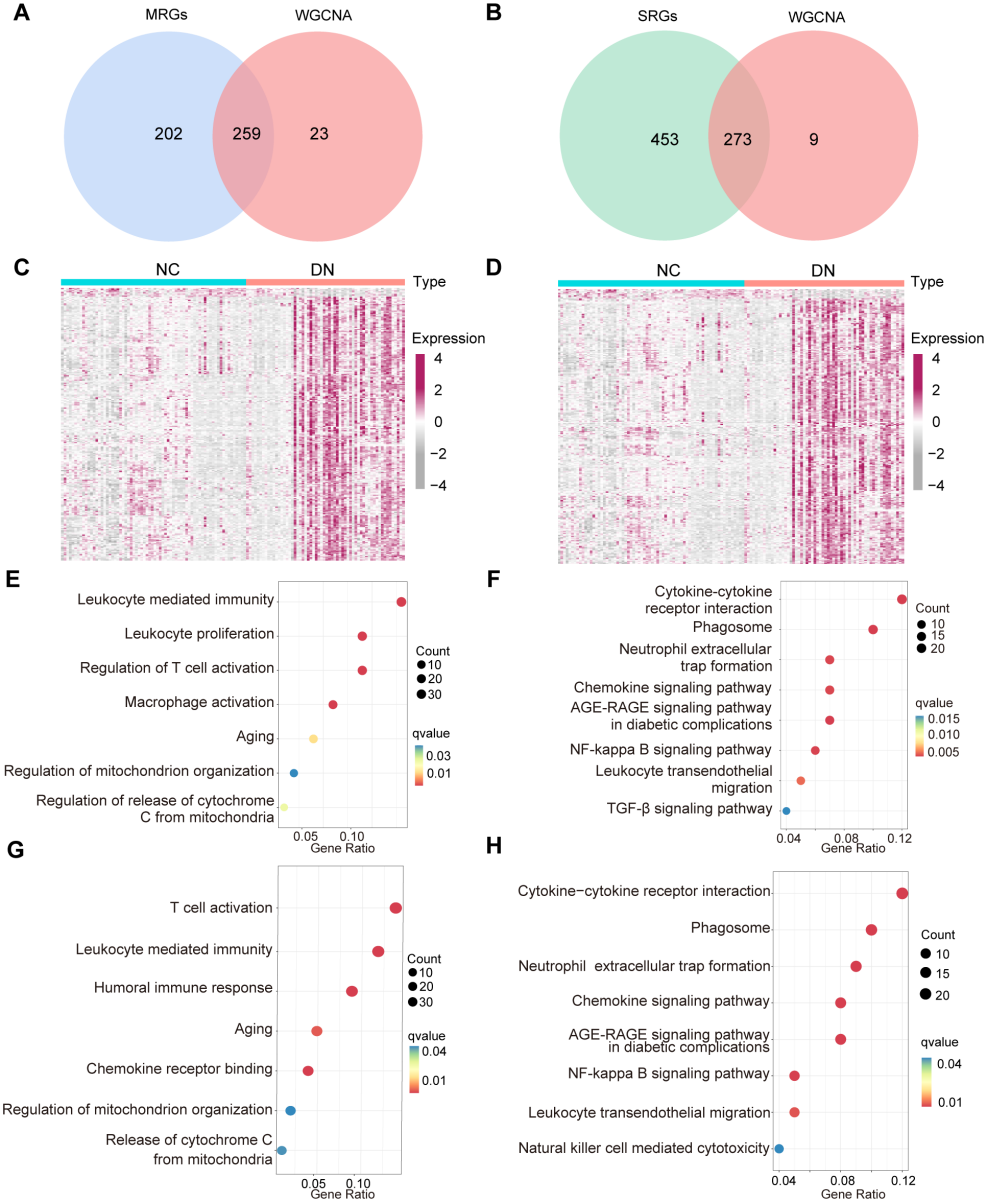
The function crossover of genes between MRGs and SRGs. **(A)** Venn diagram displaying the overlap of WGCNA model genes, and MRGs. **(B)** Venn diagram illustrating the overlap between the WGCNA model genes and SRGs. **(C)** The expression of 259 hub MRGs and **(D)** 273 hub SRGs in the training set. **(E)** GO and **(F)** KEGG enrichment results for the 259 hub MRGs, **(G)** GO and **(H)** KEGG enrichment results of 273 hub SRGs.

To explore the potential interplay between mitochondrial dysfunction and senescence in DN, we analyzed the overlap between their respective gene modules. After intersecting the above MRGs, SRGs, and DEGs, 259 hub MRGs (**Figure 5A**) and 273 hub SRGs (**Figure 5B**) were identified. Subsequently, these genes were screened between DN and NC groups by the Wilcoxon test (**Figures 5C, D**). Further exploration of gene functions was conducted through GO and KEGG pathway enrichment analyses. The GO terms enriched among the hub MRGs included T cell activation, regulation of mitochondrial organization, and leukocyte homeostasis (**Figure 5E**). Notably, KEGG pathways encompassed the AGE-RAGE signaling pathway, phagosome, and leukocyte transendothelial migration, indicating a potential association between mitochondrial dysfunction and inflammaging in DN (**Figure 5F**). Similarly, GO terms enriched for hub SRGs involved regulation of T cell activation, leukocyte chemotaxis, aging, and mitochondrial organization (**Figure 5G**). KEGG analysis further highlighted enrichment in pathways such as the AGE-RAGE signaling pathway, chemokine signaling pathway, nuclear factor-kappa B signaling pathway, and leukocyte transendothelial migration, suggesting a potential link between senescence, mitochondrial dysfunction, and inflammation in DN (**Figure 5H**). Collectively, these enrichment results demonstrated significant functional overlap between hub MRGs and hub SRGs.

**Figure 6:**
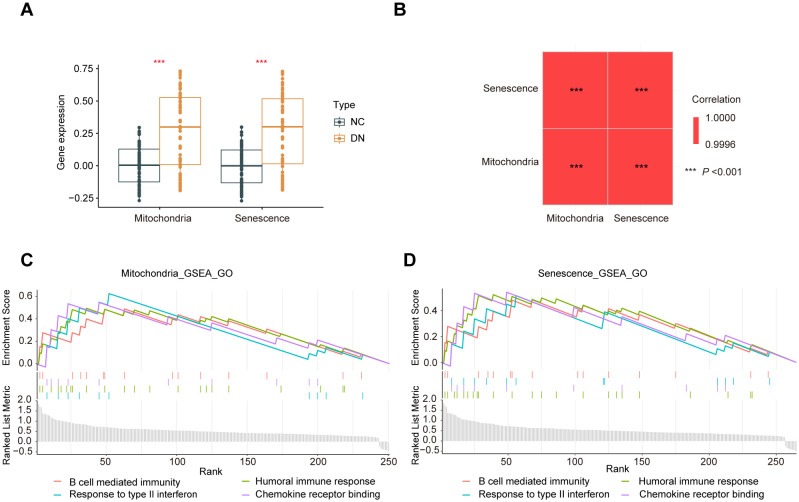
Crossover of phenotype scores between mitochondrial dysfunction and senescence. **(A)** Boxplot of phenotype scores of mitochondrial dysfunction and senescence between the DN and NC samples. **(B)** Correlation matrix of phenotype scores of mitochondrial dysfunction and senescence. **(C)** GO enrichment analysis conducted using the GSEA algorithm for the hub MRGs and **(D)** hub SRGs.

Furthermore, the ssGSEA analysis was performed to generate phenotype scores for mitochondrial dysfunction and senescence. As illustrated in **Figure 6A**, DN samples exhibited significantly higher phenotype scores for mitochondrial dysfunction and senescence (*P* < 0.001). The correlation between these two phenotype scores was remarkably strong (R > 0.9995) (**Figure 6B**). Based on these scores, hub MRGs and SRGs were divided into high- and low-expression groups. Differential expression and GSEA analyses were performed for both gene sets. Notably, mitochondrial dysfunction and increased senescence groups were significantly enriched in inflammatory pathways, such as macrophage activation, cytokine signaling, T cell activation, and chemokine responses (**Figures 6C, D**). These convergent findings further verify the strong relationship between mitochondrial dysfunction, senescence, and inflammation in the pathogenesis of DN.

### 3.4 Construction of diagnostic model for DN based on mitochondrial dysfunction and senescence using diverse machine learning approaches

**Figure 7:**
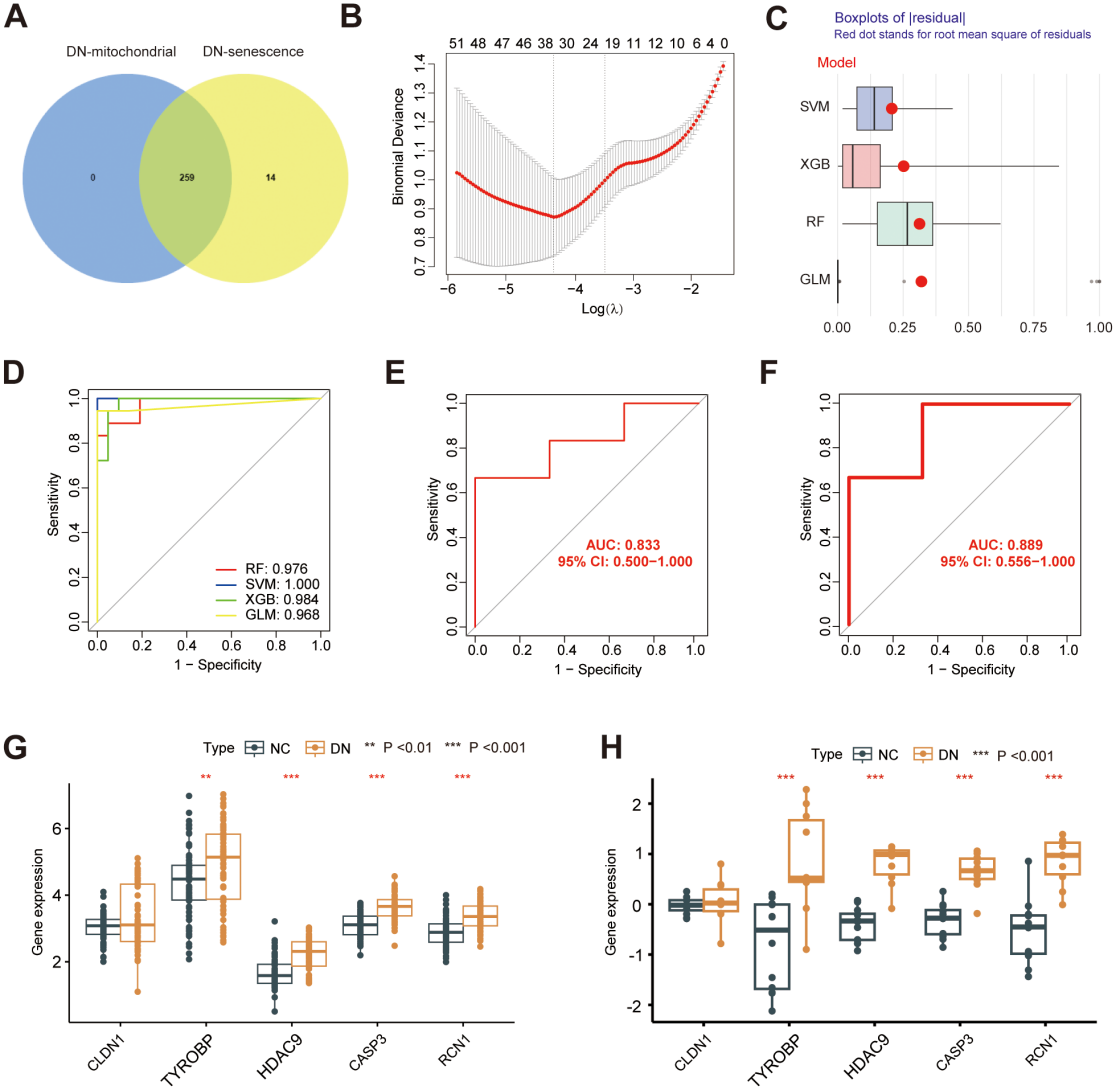
Construction of a diagnostic model. **(A)** Venn diagram showing the overlap between the hub MRG and hub SRGs. **(B)** Hub genes selection in the Lasso cox regression model. **(C)** Boxplots of residuals for each machine learning method, with RMSE marked **(D)** ROC curve of four machine learning approaches. ROC analysis and differential expression pattern of the 5-gene diagnostic model using GSE142153 **(E, G)** and GSE30529 **(F, H)** as the independent validation dataset. **P < 0.01, ***P < 0.001.

To further investigate the potential mechanisms underlying mitochondrial dysfunction and senescence in DN, we intersected hub MRGs and hub SRGs identified previously. A total of 259 shared genes were identified from this intersection (**Figure 7A**), from which key genes with potential diagnostic value for DN were screened. Of note, 34 genes were selected for Least Absolute Shrinkage and Selection Operator (LASSO) Cox regression analysis based on the minimum λ value (**Figure 7B**; **Supplementary Table 5**). Subsequently, four machine-learning approaches RF, SVM, XGB, and generalized linear model (GLM) were employed to construct diagnostic models. Cumulative residual distribution plots (**Supplementary Figure 1A**) and residual boxplots (**Figure 7C**) revealed that SVM had smaller residual values, indicating a high model accuracy. Moreover, results shown in the **Supplementary Figure 1B** illustrate the top ten variables ranked by root mean square error (RMSE) for each model. Next, the receiver operating characteristic (ROC) curves were utilized to explore the diagnostic performance of the models, with three models demonstrating excellent discriminative power, with an area under the curve (AUC) exceeding 0.95 (**Figure 7D**). Among them, SVM revealed the best predictive accuracy and reliability, making it the most suitable diagnostic model for DN. The five most significant variables in the SVM model- CLDN1, TYROBP, HDAC9, CASP3 and RCN1, were selected as the hub genes for the diagnosis of DN. The expression of the identified diagnostic genes was further verified in both GSE142153 (**Figures 7E, G**) and GSE30529 (**Figures 7F, H**) datasets, consistent with the results from the training cohort. Notably, the diagnostic model exhibited robust predictive performance in both validation sets, with AUC values exceeding 0.8. Correlation analysis revealed potential relationship between the expression of HDAC9 and SASP markers (**Supplementary Figure 2**). Furthermore, RT-qPCR was also conducted to validate the five hub genes in high glucose-stimulated HK-2 cell and NC samples (**Supplementary Figure 3**), consistent with our previous bioinformatics findings.

### 3.5 Single-cell profiling of DN

**Figure 8:**
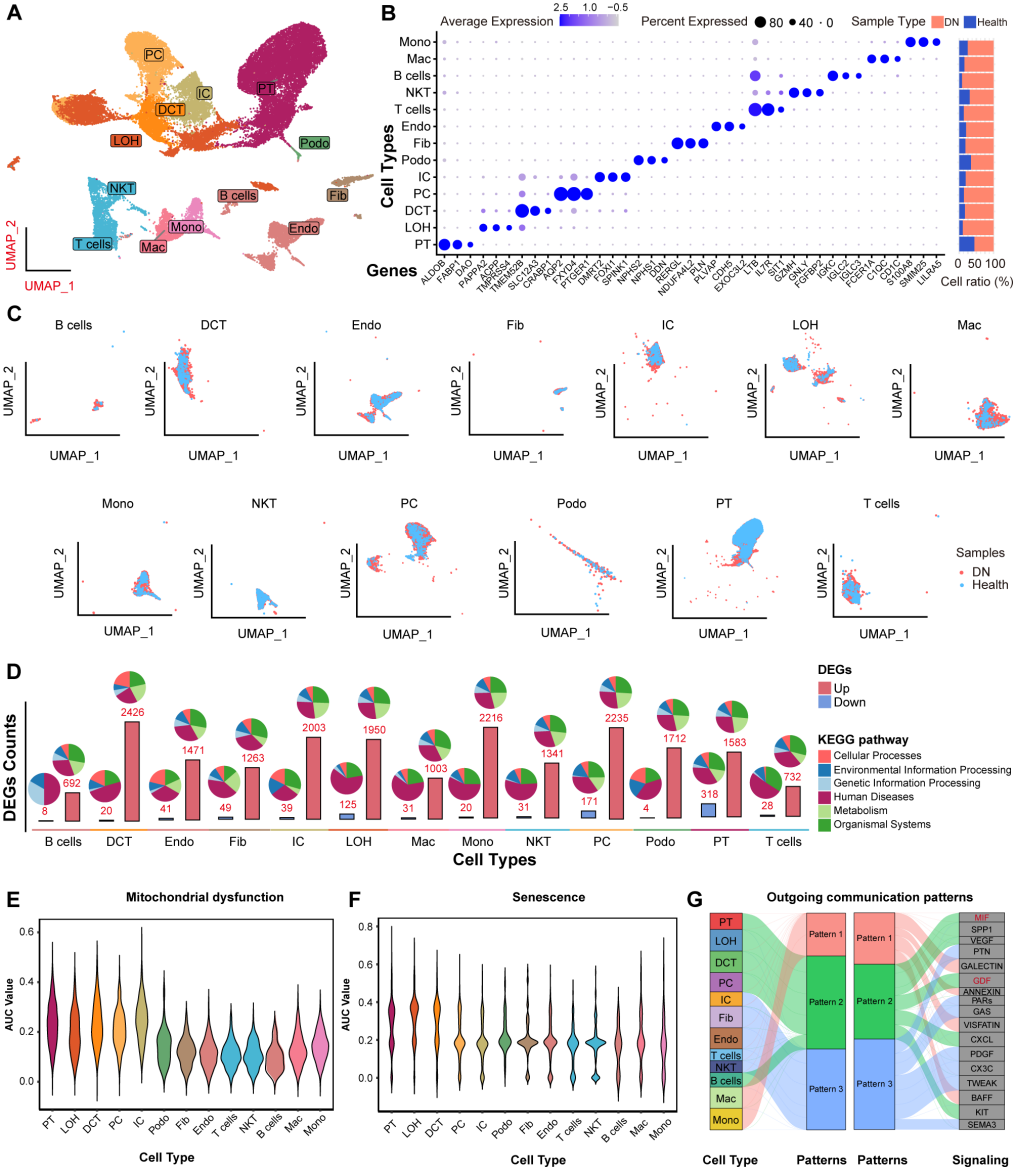
The scRNA-seq profiling of DN. **(A)** UMAP showing the 13 major cell clusters extracted from the publicly available DN scRNA-seq dataset. **(B)** Marker genes and sample origin proportions (%) for the 13 clusters. **(C)** Distribution of major cell clusters. **(D)** Barplots indicating the DEGs counts between the NC and DN samples of each cell cluster. Violin plots showing the density distribution of AUC values for **(E)** mitochondrial dysfunction and **(F)** senescence gene sets, separated by various cell types. **(G)** Alluvial plot showing the outgoing signaling patterns of target cells, also indicating the correspondence between the inferred latent patterns and cell groups, as well as signaling pathways. The thickness of the flow indicates the contribution of the cell group or signaling pathway to each latent pattern. Pathways of pattern 2 that related to mitochondrial dysfunction and senescence are marked in red.

Next, we characterized the single-cell landscape of DN. After rigorous quality control and batch effect correction, a total of 37,164 cells were yielded (**Supplementary Figure 4**). Unsupervised clustering revealed 13 distinct cell types (**Figure 8A**), including B cells, distal convoluted tubule cells (DCT), endothelial cells (Endo), fibroblasts (Fib), intercalated cells (IC), loop of Henle cells (LOH), macrophages (Mac), monocytes (Mono), natural killer T cells (NKT), principal cells (PC), podocytes (Podo), proximal tubule (PT) cells, and T cells. The cell types were annotated based on their canonical marker genes, and the relative abundance of each cell type in shown in **Figure 8B**. Notably, PT cells were the most abundant in both DN and control tissues, with a predominance of PT cells originating from DN samples. The distribution of these cell types, along with their respective tissue origins, was visualized in the UMAP plot (**Figure 8C**).

Next, we analyzed DEGs between DN and NC samples across the 13 cell types based on their transcriptional profiles. Most DEGs were linked to “human diseases” pathways. Notably, the PT cells exhibited the highest number of downregulated DEGs compared to all other cell types (**Figure 8D**, lower panel). Moreover, we investigated the activity of mitochondrial dysfunction- and senescence-related gene sets using AUCell scoring (**Figures 8E, F**). Both AUC scores were significantly elevated in PT cells, indicating that these pathways were mainly involved in DN pathogenesis, particularly within PT cells. In addition, HDAC9 expression was significantly upregulated in PT cells from DN samples (**Supplementary Figure 5**). To investigate cell-to-cell communication patterns, we applied the “CellChat” R package and identified that PT cells, LOH, DCT, PC, and B cells shared common intercellular signaling patterns in DN. Specifically, macrophage migration inhibitory factor (MIF) and growth differentiation factor (GDF) signaling pathways were enriched in pattern 2 (**Figure 8G**). Previous studies have implicated both pathways in regulating mitochondrial dysfunction and senescence (27, 28). Collectively, these findings suggest that HDAC9 may play a role in the progression of DN by regulating mitochondrial dysfunction, enhancing inflammatory responses, and promoting cellular senescence, especially in PT cells.

### 3.6 HDAC9 promotes cellular senescence and mitochondrial dysfunction

**Figure 9:**
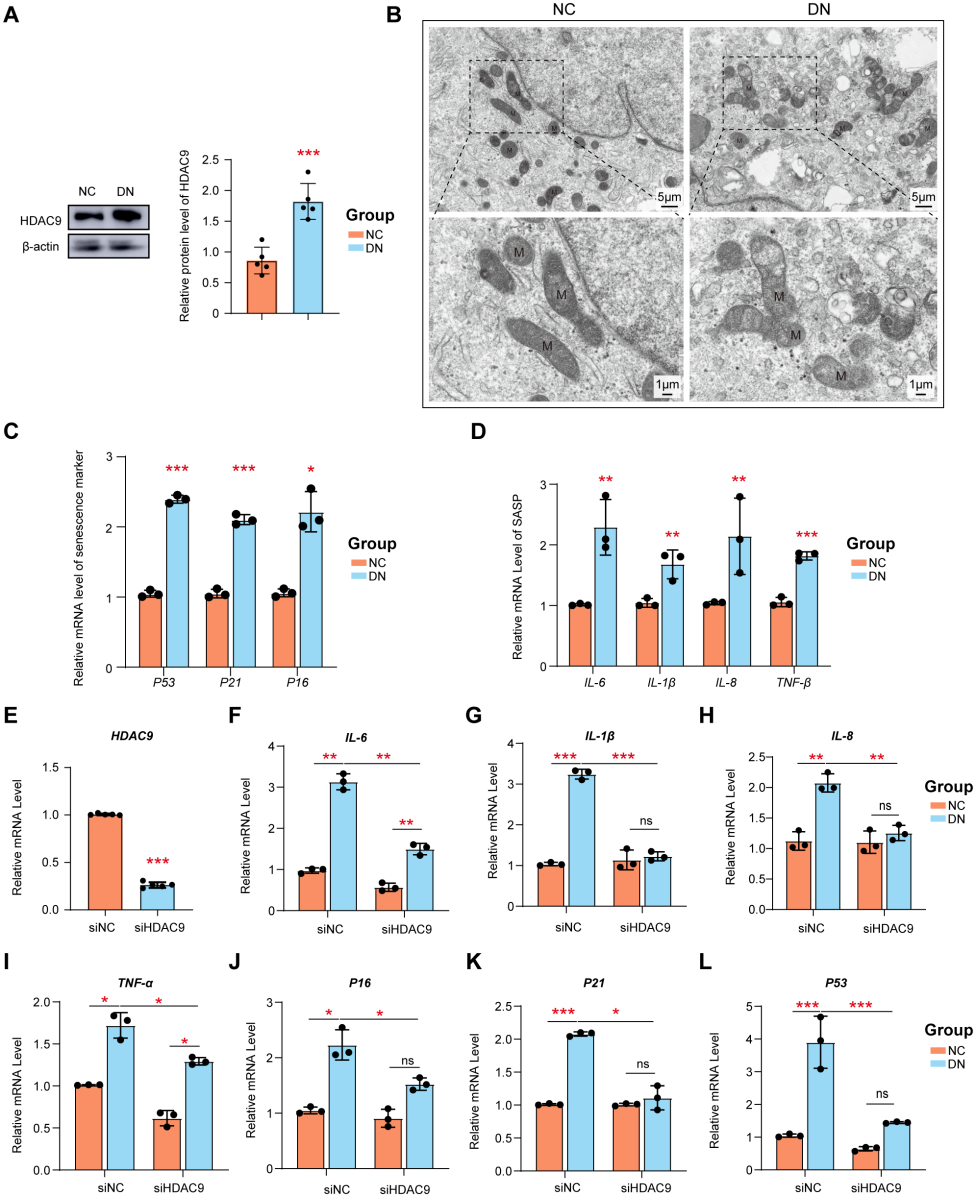
The siRNA knockdown of HDAC9 enhanced the high glucose-mediated mitochondrial dysfunction and cellular senescence. **(A)** Measurement of the protein expression of HDAC9. **(B)** Mitochondrial morphology in high glucose-mediated and normal HK-2 cells was observed by transmission electron microscope (scale bar: 5 μm and 1μm) **(C)** The relative mRNA level of p51, p21 and p16. **(D)** The relative expression of SASP was examined. **(E)** Silencing efficiency of HDAC9. The relative expression of mRNA **(F–I)** SASP and **(J–L)** p51, p21 and p16 before and after silence was examined. *: P <0.05, **: P <0.01, ***: P <0.001.

To identify the role of HDAC9 and PT cells in DN, HDAC9 inhibitor and high glucose-stimulated HK-2 cell models were used. As expected, the expression of HDAC9 was significantly upregulated in HK-2 cells following exposure to a high glucose environment compared to untreated HK-2 cells, which is in line with findings from our previous study (**Figure 9A**). As shown in **Figure 9B**, HK-2 cells exposed to high-glucose conditions exhibit significant mitochondrial structural abnormalities. These include a reduction in mitochondrial matrix density, decreased electron density, and the appearance of vacuole-like structures. Additionally, the intermembrane spaces are enlarged, and the cristae become stubby or swollen, leading to smaller, rounded mitochondria with disorganized or even absent cristae. These morphological changes reflect mitochondrial dysfunction. Senescent cells secrete various profibrotic and proinflammatory factors collectively referred to as the senescence-associated secretory phenotype (SASP). Cellular senescence plays a crucial role in the pathogenesis of DN. Established senescence markers, including p53, p21, and p16, are commonly employed to identify and characterize senescent cells (29, 30). Here, we observed a significant increase in the expression of p53, p21, and p16 (**Figure 9C**), as well as significantly upregulated mRNA levels of SASP components (IL-6, IL-1β, IL-8, and TNFα) in high glucose-stimulated HK-2 cells (**Figure 9D**). The increase in these SASP factors highlights the potential involvement of cellular senescence in DN progression.

To further verify the role of HDAC9 in DN, siRNA-mediated knockdown of HDAC9 was performed. Data shown in **Figure 9E** confirm the successful knockdown of HDAC9 expression in HK-2 cells by with a knockdown efficiency of approximately 80%. Following HDAC9 silencing, the expression levels of SASP components (IL-6, IL-1β, IL-8, and TNFα) (**Figures 9F–I**), as well as senescence markers p53, p21, and p16 (**Figures 9J–L**), were significantly downregulated in high glucose-treated HK-2 cells compared to the corresponding controls.

## 4 Discussion

Here, we identified mitochondrial dysfunction and senescence as interrelated drivers in DN. Integrative transcriptomics, WGCNA, and machine learning reveal their coordination via a shared molecular program. HDAC9 stands out as a key regulatory factor, linking mitochondrial dysfunction, cellular senescence signaling, and inflammatory activation, with pronounced effects observed within PT cells.

Kidneys are characterized by a high mitochondrial density, with the second-highest oxygen consumption rate in the body at rest (31). Most oxygen is utilized through oxidative phosphorylation (OXPHOS) in the mitochondria, which drives ATP synthesis. Approximately 90% of renal ATP is generated via OXPHOS under physiological conditions, ensuring efficient ATP production (32, 33). ATP primarily supports epithelial cells in actively reabsorbing sodium, glucose, ions, and other metabolites from the filtrate, with PT cells playing a predominant role. These cells comprise about 90% of the outer renal cortex and approximately 50% of total kidney mass. The development of DN implicates renal cells with high metabolic demands and strong dependence on mitochondrial ATP production, particularly PT cells. Due to their high reliance on mitochondrial ATP production, PT cells are especially susceptible to metabolic stress, contributing to their functional deterioration in DN. This inherent vulnerability may underlie the observed HDAC9-mediated disruption of mitochondrial dynamics and further supports the role of mitochondrial dysfunction as a key driver of senescence in diabetic kidneys.

However, hyperglycemia-driven overproduction of ROS, including superoxide, hydrogen peroxide, and peroxynitrite, induces oxidative damage to mitochondrial proteins and mitochondrial DNA (mtDNA), ultimately impairing mitochondrial function. Elevated ROS levels that exceed the local antioxidant defenses are considered hallmarks of mitochondrial dysfunction in DN (34–36). Although these associations are well established, the precise contributions of specific ROS species to the pathogenesis of DN are not well understood. Moreover, identification of novel biomarkers associated with the mitochondrial dysfunction may reveal important mechanistic insights and improve early diagnosis.

The progression process of PT cells in response to DN is a complex, multistep process involving cellular proliferation, cell cycle arrest and hypertrophy (37). Over time, these maladaptive responses culminate in a terminal phenotype marked by molecular signatures of cellular senescence, which not only indicate irreversible growth arrest but also drive chronic inflammation and tissue remodeling. The terminal stages of this process are characterized by a molecular signature associated with the cellular senescence. Senescence, a process linked to age-related diseases such as DM (38), plays a critical role in the progression of DN (24, 39, 40). Increasing evidence suggests that inflammation is more strongly associated with kidney senescence than with infectious or toxic causes (41). Importantly, recent studies have emphasized the central role of mitochondrial dysfunction in promoting renal ageing and chronic kidney disease (CKD). Mitochondrial abnormalities—such as impaired oxidative phosphorylation, increased mitochondrial ROS production, and decreased mitochondrial biogenesis—are widely observed in CKD and are closely associated with PT cell senescence, inflammation, and fibrosis (9, 12, 13, 42). Additionally, mitochondrial dysfunction not only disrupts key metabolic pathways—including tryptophan and bile acid metabolism—but also activates profibrotic signaling cascades such as TGF-β, thereby accelerating CKD progression (34, 35). These mitochondrial impairments not only drive intrinsic tubular injury but also trigger maladaptive immune responses, creating a vicious cycle of inflammation and damage in the senescent kidney (10, 36). Given the clinical and mechanistic overlap between CKD and DN, these mitochondrial abnormalities are likely to be exacerbated in the diabetic kidney. In DN, high glucose levels and metabolic stress further compromise mitochondrial integrity, leading to mtDNA damage, ROS accumulation, and altered mitochondrial dynamics. These perturbations reinforce senescence-associated phenotypes and chronic inflammation, underscoring mitochondrial dysfunction as a pathogenic nexus linking cellular senescence and DN progression. Thus, targeting HDAC9 may offer new therapeutic avenues for DN.

HDAC9, a class IIa histone deacetylase, has been implicated in various processes, including lipid metabolism, atherosclerosis progression, and macrophage polarization (43). In the kidney, HDAC9 contributes to development of podocyte injury and renal damage in diabetic conditions (44). However, the role of HDAC9 as a universal factor in kidney senescence across different CKD conditions remain unclear, and its specific involvement in DN progression has not been thoroughly explored. Senescence is now recognized as a major contributor to the onset and progression of CKD, acting through mechanisms such as mitochondrial dysfunction, chronic inflammation, and metabolic dysregulation. Emerging evidence has identified altered tryptophan and bile acid metabolism (45, 46), as well as TGF-β—driven fibrotic signaling (47), as key senescence-related mechanisms contributing to renal pathophysiology. Moreover, integrative multi-omics studies have revealed robust senescence-associated signatures and immune alterations in DN (21, 22, 48), highlighting their potential as diagnostic markers and therapeutic targets. Pharmacological interventions targeting senescence, including natural compounds like Alpiniae oxyphyllae fructus (49), are also being explored as promising strategies to mitigate senescence-related renal injury. These findings delineate senescence-associated mechanisms in CKD and lay the groundwork for investigating how regulators like HDAC9 contribute to DN. The present study demonstrates for the first time that elevated expression of HDAC9 significantly induces mitochondrial dysfunction and senescence, which in turn promotes the progression of DN. Collectively, as our understanding of these mechanism advances, HDAC9 may not only serve as a prognostic biomarker but could also be targeted therapeutically for DN treatment, although this requires continued research.

Importantly, these findings also carry notable clinical implications. HDAC9 expression could serve not only as a potential biomarker for early detection of DN but also as a stratification factor to identify patients more likely to benefit from mitochondria-targeted or senescence-modulating therapies. Given that mitochondrial dysfunction and chronic inflammation are hallmark features of progressive DN, interventions aimed at restoring mitochondrial homeostasis or attenuating SASP may improve renal outcomes. Furthermore, the upregulated expression of HDAC9 in PT cells—central players in DN pathophysiology—suggests that HDAC9 inhibition may provide cell-type-specific therapeutic advantages. Future clinical studies should evaluate whether HDAC9 levels in renal biopsy specimens or noninvasive samples (e.g., urine exosomes) correlate with disease severity, prognosis, or treatment response in diabetic patients.

Nonetheless, this study has certain limitations. First, although HDAC9 knockdown significantly reduced senescence and inflammatory markers *in vitro*, the exact molecular targets and pathways via which HDAC9 modulates mitochondrial dynamics were not clarified. Second, this investigation was based on transcriptomic data; integration with proteomic and metabolomic datasets would strengthen causal inferences. Third, due to missing clinical information in public DN datasets, multivariate statistical analyses related to prognosis or treatment response could not be performed. Future studies with well-annotated patient cohorts will be essential for such integrative analyses. Finally, while HK-2 cells provide a convenient model, future *in vivo* experiments using in diabetic mouse models are advocated to validate therapeutic targeting of HDAC9. Addressing these limitations in future studies will be crucial for translating our findings into clinical applications, which may ultimately lead to improved therapeutic approaches for patients with DN.

In summary, HDAC9 inhibition may simultaneously alleviate mitochondrial dysfunction, senescence, and inflammation, providing a potential therapeutic strategy for DN.

## 5 Conclusion

In summary, this study highlights the critical role of the key genes associated with mitochondrial dysfunction and senescence, demonstrating their potential as diagnostic biomarkers for DN. Moreover, targeting HDAC9 may offer a novel therapeutic approach by ameliorating mitochondrial damage, senescence, and inflammation in DN. These insights provide a new conceptual framework for understanding DN pathogenesis and propose novel avenues for developing therapeutic intervention targeting the mitochondria-senescence-inflammation axis.

## Data Availability

The datasets presented in this study can be found in online repositories. The names of the repository/repositories and accession number(s) can be found in the article/**Supplementary Material**.

## Supplementary material

Supplementary Figure 1 Performance Comparison of Machine Learning Models. **(A)**The top ten variables ranked by the RMSE for each model. **(B)** Cumulative residual distribution maps of the four approaches.

Supplementary Figure 2 Correlation analysis between the expression of HDAC9 and SASP markers in kidney-derived samples in GSE30529.

Supplementary Figure 3 The RT-qPCR validation of CLDN1, TYROBP, HDAC9, CASP3 and RCN1 expression between high glucose-stimulated HK-2 cell and healthy samples.

Supplementary Figure 4 Quality control of scRNA-seq data.

Supplementary Figure 5 Expression profiles of five model genes in different renal cell types in DN and NC samples. Violin plots showing the expression levels of the five model genes in various cell types from DN and NC samples. Statistical significance was assessed using the Wilcoxon rank-sum test. *: *P* < 0.05, ***: P* < 0.01, ***: *P* < 0.001.

Supplementary Table 1 The primers used in the study.

Supplementary Table 2 The antibodies used in the study.

Supplementary Table 3 List of MRGs.

Supplementary Table 4 List of SRGs.

Supplementary Table 5 List of 34 hub genes after Lasso cox regression.
