# Supplementary figures and images for: Integrative transcriptomic and machine learning analyses identify HDAC9 as a key regulator of mitochondrial dysfunction and senescence-associated inflammation in diabetic nephropathy
Source: Front Immunol. 2025 Aug 29;16:1627173. doi: 10.3389/fimmu.2025.1627173 (PMC12425722; doi:10.3389/fimmu.2025.1627173)

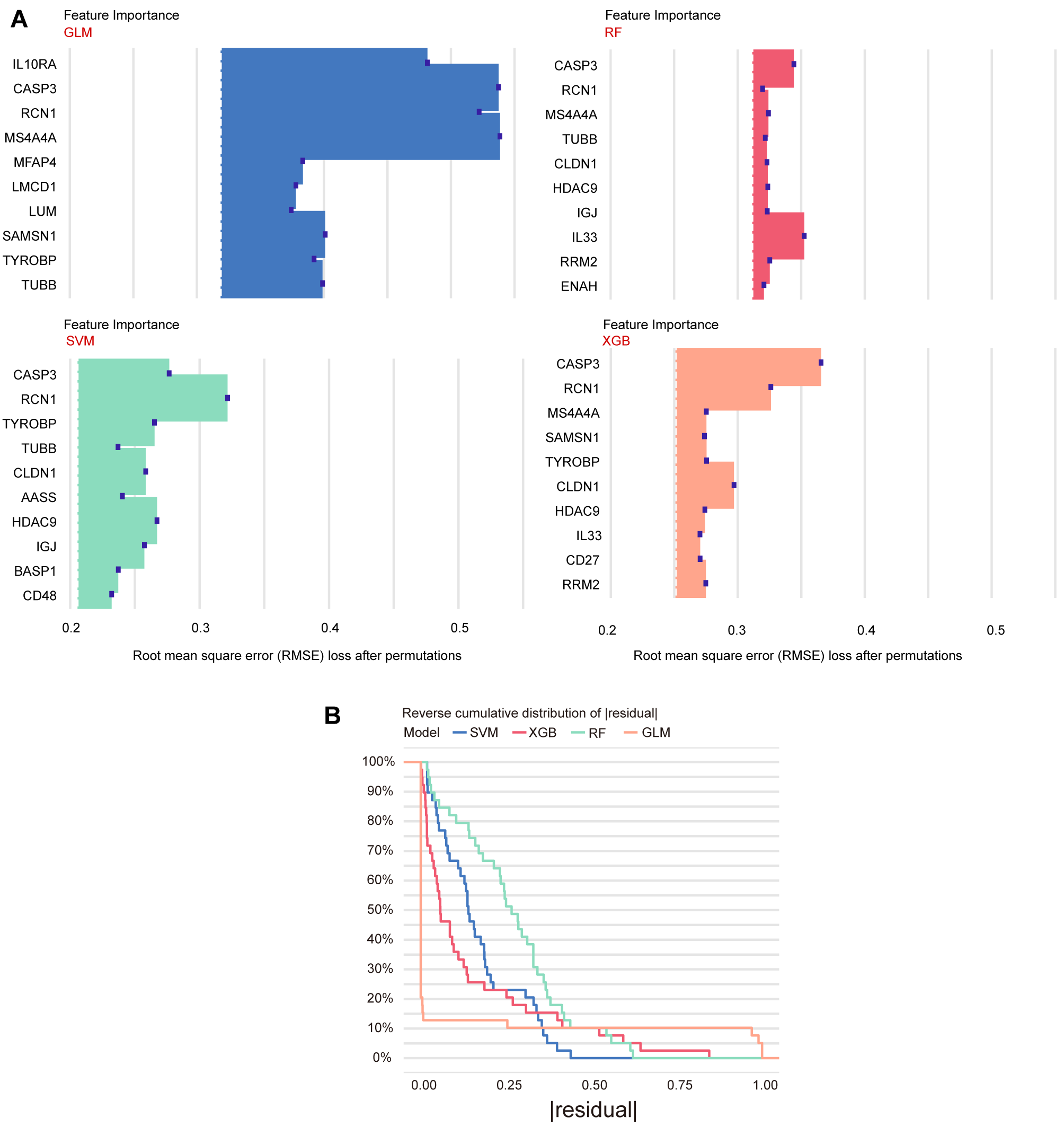

Supplement: Supplementary Figure 1 — Performance Comparison of Machine Learning Models. (A)The top ten variables ranked by the RMSE for each model. (B) Cumulative residual distribution maps of the four approaches. [file Image1.tif]

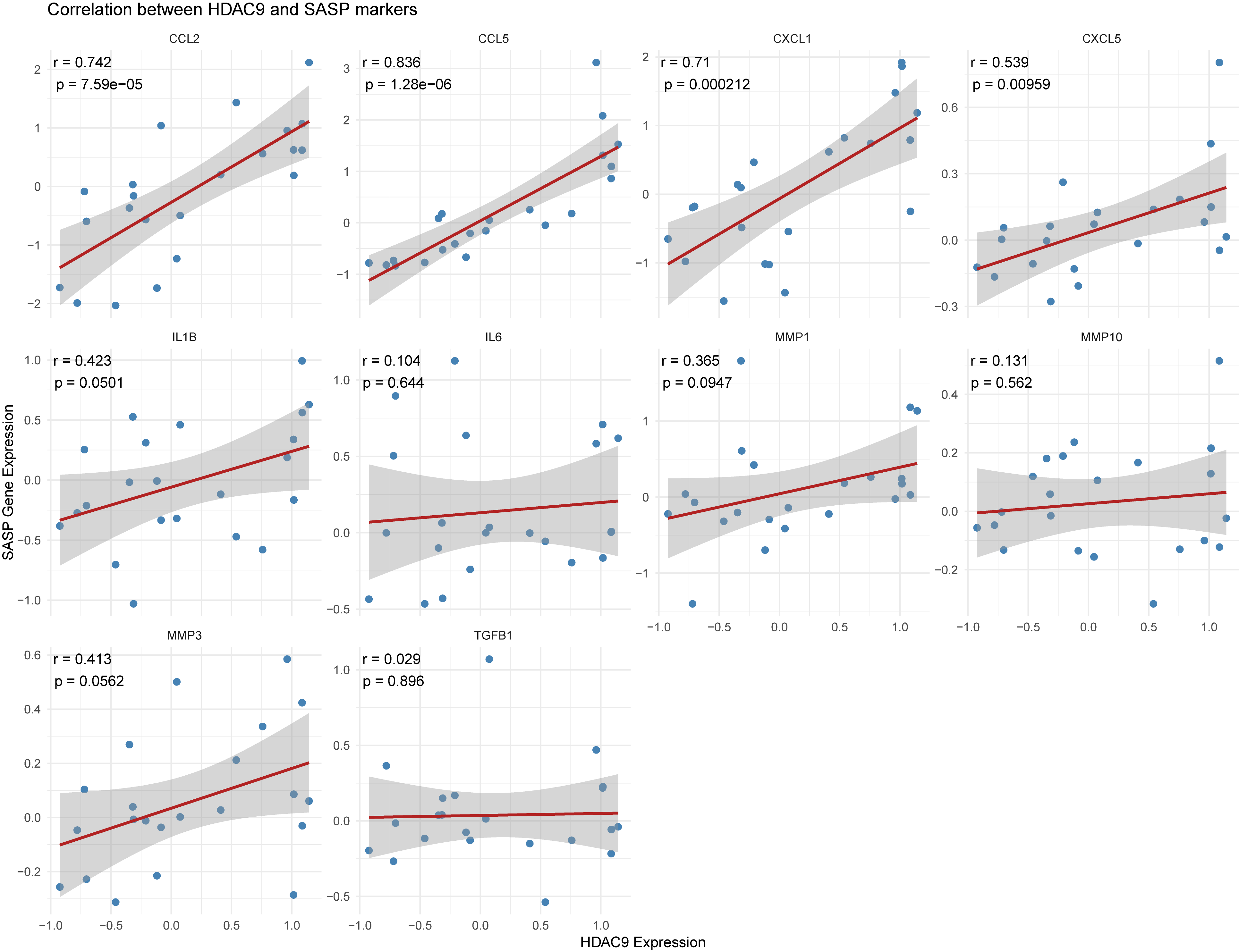

Supplement: Supplementary Figure 2 — Correlation analysis between the expression of HDAC9 and SASP markers in kidney-derived samples in GSE30529. [file Image2.tif]

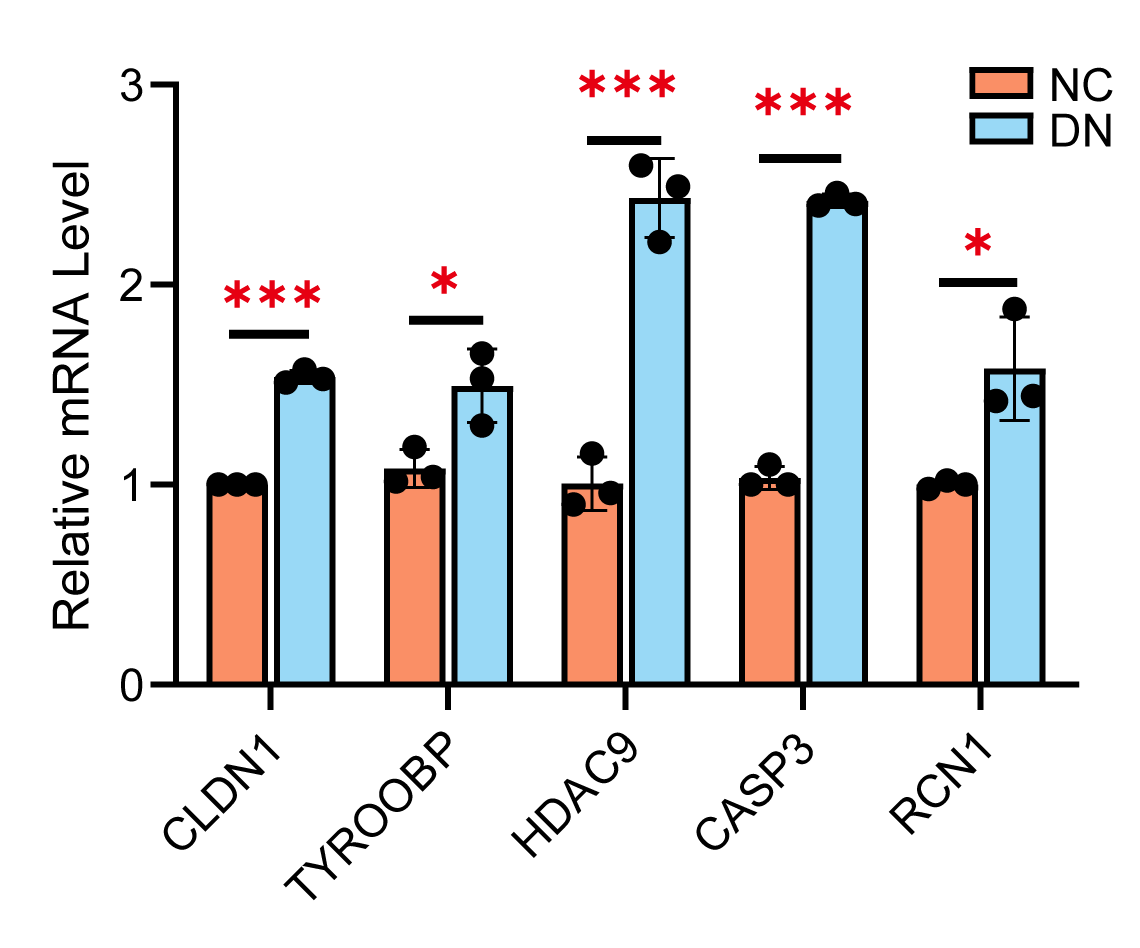

Supplement: Supplementary Figure 3 — The RT-qPCR validation of CLDN1, TYROBP, HDAC9, CASP3 and RCN1 expression between high glucose-stimulated HK-2 cell and healthy samples. [file Image3.tif]

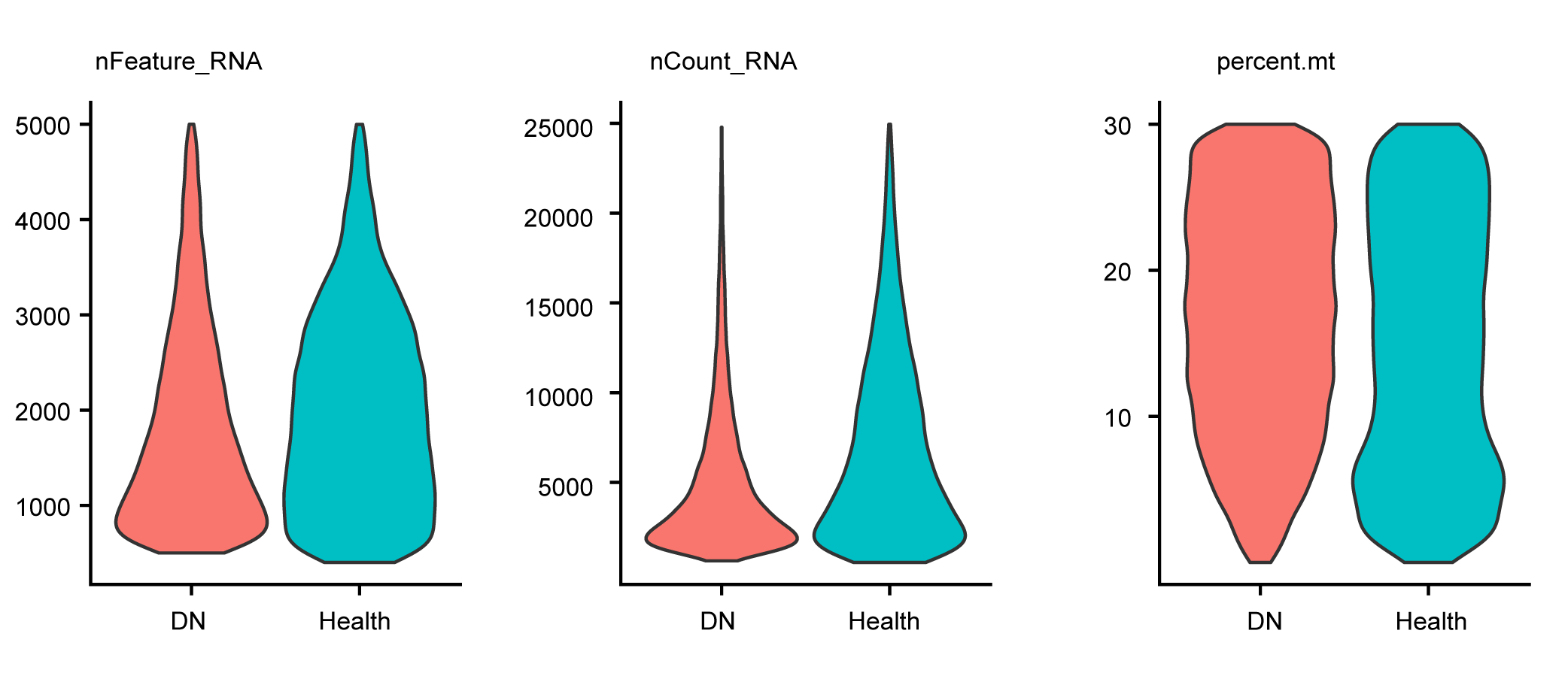

Supplement: Supplementary Figure 4 — Quality control of scRNA-seq data. [file Image4.tif]

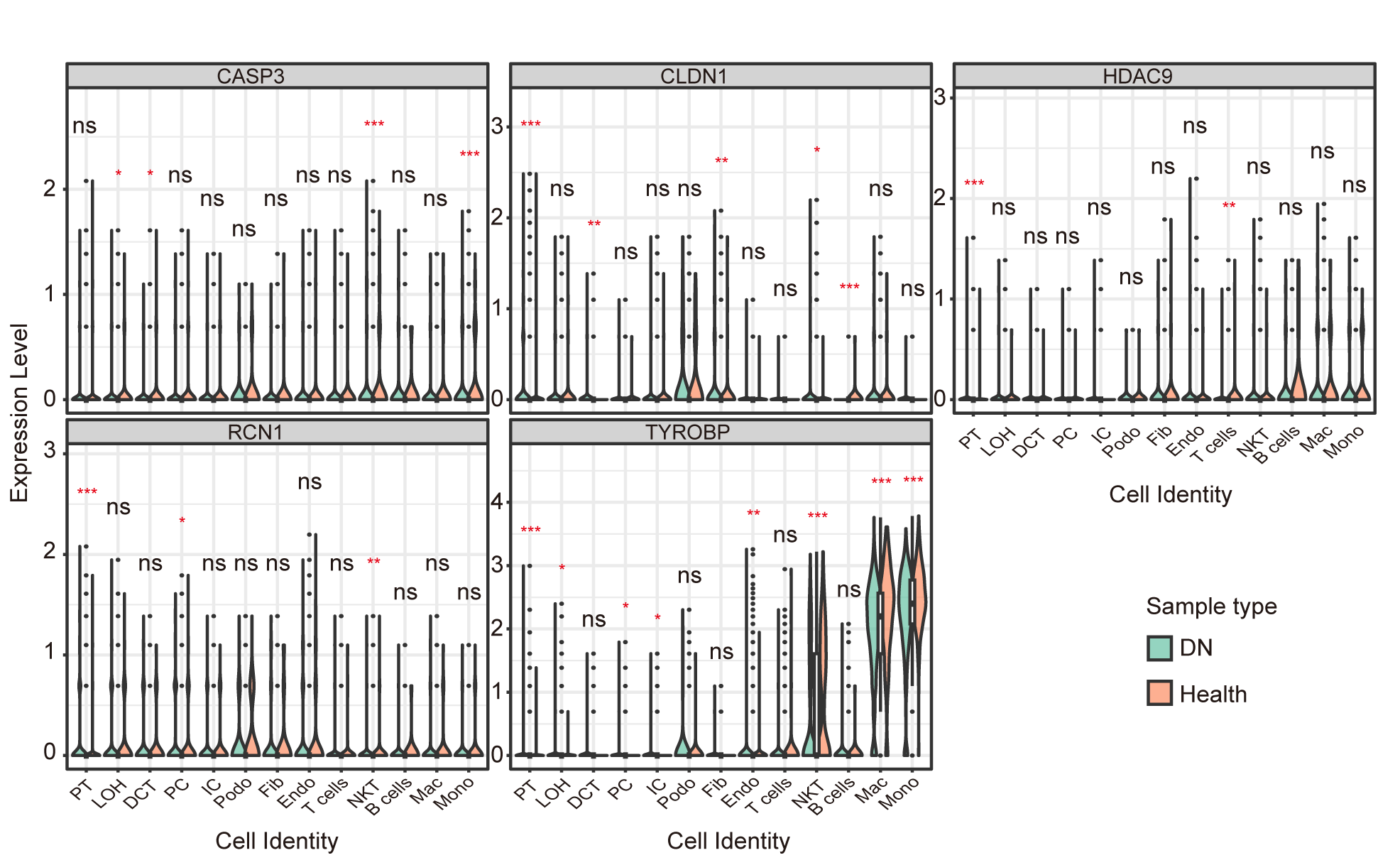

Supplement: Supplementary Figure 5 — Expression profiles of five model genes in different renal cell types in DN and NC samples. Violin plots showing the expression levels of the five model genes in various cell types from DN and NC samples. Statistical significance was assessed using the Wilcoxon rank-sum test. *: P < 0.05, **: P < 0.01, ***: P < 0.001. [file Image5.tif]
